# Community-based participatory research (CBPR) approaches in vaccination promotion: a scoping review

**DOI:** 10.1186/s12939-024-02278-1

**Published:** 2024-11-05

**Authors:** Yan Zhang, Yao Jie Xie, Lin Yang, Kin Cheung, Qingpeng Zhang, Yan Li, Chun Hao, Harry HX Wang, Qianling Zhou, Angela Yee Man Leung

**Affiliations:** 1 School of Nursing, Faculty of Health and Social Sciences, The Hong Kong Polytechnic University, Kowloon, Hong Kong SAR China; 2 Cardiology Department, The Affiliated Hospital of Qingdao University, Qingdao, China; 3 Research Centre for Chinese Medicine Innovation, The Hong Kong Polytechnic University, Kowloon, Hong Kong SAR China; 4 Musketeers Foundation Institute of Data Science, The University of Hong Kong, Pok Fu Lam, Hong Kong SAR China; 5 Department of Pharmacology and Pharmacy, LKS Faculty of Medicine, The University of Hong Kong, Pok Fu Lam, Hong Kong SAR China; 6 Department of Medical Statistics, School of Public Health, Sun Yat-sen University, Guangzhou, China; 7 Usher Institute, Deanery of Molecular, Genetic & Population Health Sciences, The University of Edinburgh, Edinburgh, UK; 8 Department of Maternal and Child Health, School of Public Health, Peking University, Beijing, China; 9 Research Institute for Smart Aging, The Hong Kong Polytechnic University, Kowloon, Hong Kong SAR China; 10 Research Centre of Textile for Future Fashion, The Hong Kong Polytechnic University, Kowloon, Hong Kong SAR, China; 11 WHO Collaborating Centre for Community Health Service, School of Nursing, The Hong Kong Polytechnic University, Kowloon, Hong Kong SAR, China

**Keywords:** Community-based participatory research, Community-academic partnership, Community-based intervention, Scoping review, Vaccination promotion

## Abstract

**Background:**

Community-based participatory research (CBPR) is a collaborative research approach that engages academic researchers and community stakeholders as equal partners in all research steps to address community concerns and achieve health equity. The CBPR approach has been widely used in vaccination promotion programmes. However, the elements and steps of CBPR-based programmes varied among studies. The purpose of this scoping review was to synthesize the elements and steps, and establish an implementation framework to guide the utilisation of CBPR approaches in vaccination promotion.

**Methods:**

This scoping review was performed in accordance with Arksey and O’Malley’s five-stage framework. A systematic search was conducted on a set of electronic databases and grey literature sources. The retrieved articles were screened according to the criteria of CBPR and vaccination promotion, and data were extracted and recorded on a calibrated and predefined form in terms of study characteristics and CBPR components. Two authors worked independently to complete literature search, study selection, and data extraction. A narrative summary was used in categorising characteristics, and the contents of the included studies were summarised through qualitative analysis.

**Results:**

A total of 8557 publications were initially screened, and 23 articles were finally included. According to the CBPR conceptual model, the elements in each CBPR component specifically for vaccination promotion included (1) the establishment of community–academic partnership (CAP)s, (2) community capacity building by partner training vaccination knowledge, research literacy, and service abilities and skills, (3) development and implementation of community-based intervention and (4) Outcome evaluation. A CAP was established between academic researchers or institutes and eight types of partners, including community service organisation–related non-government organisations (NGOs), health service institution–related NGOs, religious organisations, government agencies, educational institutions, media agencies, business agencies, and community representatives. The maintenance of CAP was achieved with four key strategies, namely, strengthening communication, forming management groups, sharing resources and information, and providing incentives. Twelve studies provided comprehensive insights into the strategies employed for intervention development, utilising either quantitative surveys, qualitative methods or a combination of both approaches. The contents of interventions included health service supports, health education activities, social marketing campaigns, community mobilisation, interactive discussions, vaccination reminders and incentives. As for outcome evaluation, vaccination rate and the effectiveness of interventions were assessed. A considerable increase was observed in 95.7% of the included studies (22/23), and the highest increase (92.9%) was attained after the intervention. An implementation framework was generated to summarise the elements and steps of CBPR approaches for vaccination promotion.

**Conclusions:**

This review summarised current evidence and generated an implementation framework to elucidate the elements and steps in the development and application of CBPR approaches in vaccination promotion. CBPR approaches are recommended for future vaccination promotion programmes, involving community stakeholders and research professionals, to ensure equitable access to vaccinations across diverse populations.

**Supplementary Information:**

The online version contains supplementary material available at 10.1186/s12939-024-02278-1.

## Background

The mass distribution of vaccines is one of the greatest public health achievements in history and has reduced morbidity, disability and mortality due to various infectious diseases worldwide [1]. The routine childhood immunisation schedule of the 2009 birth cohort in the United States has prevented approximately 42,000 early deaths and 20 million diseases, saving $13.5 billion in direct cost and $68.8 billion in total cost [2]. The Global Vaccine Action Plan 2011–2020 (GVAP) set forth various vaccination targets, such as poliomyelitis, measles, diphtheria, tetanus, and pertussis, and has proposed a coherent global framework for immunisation [3]. Building upon the GVAP and unmet vaccination targets, the Immunization Agenda 2030 (IA 2030) called for equitable access to routine vaccines, aiming to ensure that all people benefit from recommended immunisations throughout the life course [4]. However, the immunisation coverage of many vaccines has not reach the expected level. For example, a systematic analysis examined the coverage of routine childhood vaccination in 204 countries from 1980 to 2019 and demonstrated that global vaccine coverage broadly plateaued over the past decade, with only 11 countries reaching the 90% coverage target for all vaccines in 2019 [5]. Moreover, a pooled analysis quantified the worldwide cumulative coverage of human papillomavirus (HPV) vaccination, indicating that the full course of HPV vaccine in low-income or lower-middle-income countries had a total population coverage of 1.4%, which was far below the threshold of 70% HPV vaccination coverage in developed countries [6]. With the end of the GVAP era and the start of the IA 2030 plan, the COVID-19 pandemic presented further challenges to routine immunisation throughout the world in 2020. Academic wisdom and practical experience converges to reach GVAP targets and IA 2030 ambitions, and participatory research emerges as the most accepted and recognised response [7].

Participatory research serves as an umbrella term that covers a variety of interrelated research methods, such as community–academic partnership (CAP), participatory action research, community-engaged research and community-based participatory research (CBPR) [8, 9]. CBPR originated from public health research in the 1990s and has become the gold standard for participatory research. CBPR not only recognises the inherent complexity of health disparities and the importance of incorporating diverse perspectives, but it also utilises tools and methodologies from various disciplines to comprehensively understand and analyse the multifaceted forces that contribute to these disparities [10]. This methodology fosters dialogue and joint decision-making among various stakeholders within the community, aiming to ensure equitable allocation of resources, service provision, and health policy development. Previous studies indicated that utilizing CBPR as a collaborative process helped individuals understand the significant system-level changes necessary to address disparities and inequities, and it has been established within health inequity research [11, 12]. It differs from other participatory research methods and is a collaborative approach that equalises power relationship between academic researchers and community stakeholders, which is an orientation to research that involve community in several phages of research process, such as issue identification, data collection, outcome evaluation, and result dissemination [13]. The role of each partner is defined at the start of a research project [14]. CBPR is characterised by the highest level of community participation on the continuum developed by the National Center for Research Resources at the National Institutes of Health, progressing from outreach to involvement and empowerment [15]. Notably, CBPR is a community-driven research paradigm that fits well with community needs to improve health and reduce disparity [16]. A conceptual CBPR model based on extensive literature reviews, CBPR practitioner surveys, and CBPR expert consultations was developed with four major domains: context, partnership dynamics, research, intervention and outcome [17]. This conceptual model has been utilised in CBPR programmes, but its application within the realm of vaccination promotion requires further enhancement.

CBPR takes predominance in global immunisation strategies, showing potential capacity to boost vaccination equity. One proposed strategy to mitigate disparity associated with vaccines is to prioritize vaccination coverage. Previous studies have achieved preliminary progress in addressing substantial disparities in vaccine-preventable diseases by increasing vaccination rates in different populations. For instance, a study reported that immigrants/migrants such as refugees, asylum seekers, and individuals without legal documentation expressed increased motivation to receive COVID-19 vaccine after a CBPR-based intervention [18]. A preliminary study showed that CBPR intervention increased HPV vaccination rate to 92.9% in 323 Peruvian female adolescents [19]. Another large-scale study in Pakistan demonstrated that CBPR intervention achieved a good vaccination rate (74%) for two typhoid fever vaccines administered to 21,059 children aged 2–16 years [20]. However, owing to the lack of unified theoretical guidance of CBPR approach in vaccination promotion, substantial discrepancy in practical application has emerged among various CBPR programmes. A uniform framework is required to standardise the application of CBPR approaches in boosting vaccination. Therefore, this scoping review was performed to summarise the elements and steps of CBPR approaches and to formulate an implementation framework that can guide the utilisation of CBPR approaches in vaccination promotion.

## Methods

This scoping review was performed in accordance with the five-stage methodological framework for scoping reviews defined by Arksey and O’Malley (2005) [21] and refined by Levac et al. (2010) [22]. This five-stage methodological framework included specifying research question, identifying relevant studies, selecting eligible studies, charting data and collating, summarising and reporting results [23, 24]. This scoping review was conducted in accordance with the Preferred Reporting Items for Systematic Reviews and Meta-analyses extension for scoping reviews (Supplementary Material Table S1).

### Step 1. Specifying research question

Two research questions were addressed in this scoping review: how the CBPR approach was used in community-based vaccination promotion programmes, that is, what were the key elements of CBPR in the vaccination promotion programmes, such as types of community partners, participation phases, and strategies for establishing and maintaining a CAP; community partners training components; development and implementation of the programmes; and outcome evaluation. Another question was how were these elements organised together to effectively increase the vaccine uptake for community residents.

### Step 2. Identifying relevant studies

A systematic literature search was conducted to identify peer-reviewed publications and grey literature. Firstly, an initial search was conducted in PubMed to find articles related to the topic, and the keywords CBPR and vaccination were identified in the title, abstract and index of papers. Then, using the identified search terms, consisting of Medical Subject Headings terms and keywords on concepts of CBPR and vaccination, the formal search was conducted in the indexed databases including PubMed, Embase, Web of Science, and Cochrane Library. The search strategy was modified based on the specification of each database (Supplementary Material Table S2).

Google and Google Scholar were searched for grey literature. The reference lists of the retrieved publications were manually searched for additional relevant literature. The systematic and grey literature search incorporated publication dates from database inception to January 2024. The species filter was limited to ‘Humans’, and no other restrictions were imposed on language, population, and study design.

### Step 3. Selecting eligible studies

Eligible studies for this scoping review were original publications using CBPR approaches for vaccination promotion. The PICOS strategy, consisting of population, interventions, comparisons, outcomes and study design, was used in searching the literature.

Population (P): The study population covered all age groups which spanned from children to adults. Intervention (I): The eligible intervention was the CBPR approach. The operational definition of CBPR in the study selection process involved the active engagement of community stakeholders as the partners of researchers [25]. Community and academic partners collaborated at a minimum of two phases of the research process, including subject recruitment, intervention development, intervention delivery, data collection, results interpretation, and dissemination. For example, community partners had influence in the selection of research topics, research decision-making, data collection or research results interpretation and dissemination [23, 24]. Comparison (C): Eligible comparison included blank control, active control or any other interventions without community participation in the control group intervention. No predefined limitations were imposed on the pre-post design. Outcome (O): The primary outcome was the vaccination rate for any recommended vaccine for children, adolescents and adults. Study design (S): The original study with an experimental design in terms of randomised controlled trials (RCT), cluster RCT, non-RCT and pre-post design was eligible. Qualitative studies, case reports, conference presentations, study protocols, editorials, commentaries, perspectives, letters, and abstracts were excluded.

Two authors independently assessed the studies for inclusion and exclusion through a sequential process involving title, abstract, and full-text screening. Any disagreements were discussed until a consensus was reached.

### Step 4. Charting data

Initially, a standardised data-charting form was created, and then a pilot test was performed by two reviewers, who assessed the three included papers to ensure consistency. After necessary adjustments, the standardised form was used for data extraction. Two authors extracted data, including the types of partners and their participation phases, contents of community partner training and community-based interventions involved in each study. In case of discrepancies between the reviewers, a third reviewer was consulted, who was responsible for the entire review process.

### Step 5. Collating, summarising and reporting results

A narrative summary of the characteristics of these studies was made, and the contents of the literature were summarised through qualitative analysis. A widely used conceptual model of CBPR was used in the formulation of a CBPR-based implementation framework for vaccination promotion, which contained four domains: context, partnership dynamics, research, and intervention and outcome [17]. As the studies included in our scoping review did not provide sufficient information about the context of vaccination promotion programmes, this scoping review only focused on the other three domains to summarise the application of CBPR approaches. The key results were reported in four aspects: establishing CAP, building community capacity, developing and implementing community-based interventions and evaluating outcomes. An adaptive CBPR-based implementation framework for vaccination promotion was generated according to the three domains in the CBPR conceptual model and four elements in CBPR programmes.

## Results

### Selection of studies

**Fig. 1:**
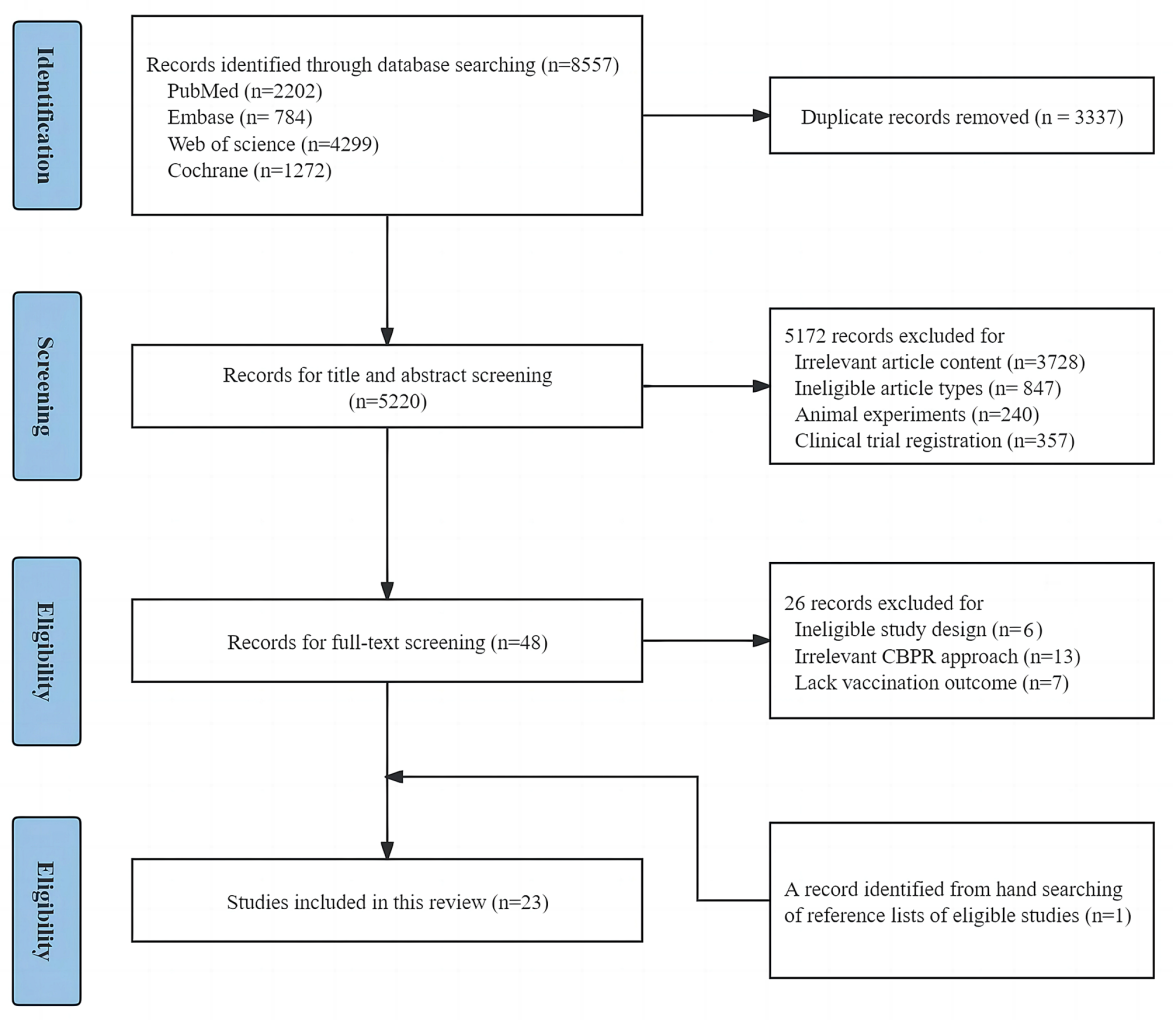
Flowchart diagram of study selection

Figure 1 presents the flowchart of the study selection process. The literature search identified 8557 records, and 8535 of the articles were excluded because of irrelevant article contents, ineligible study design, animal experiments and non-CBPR approaches. A total of 22 studies met the outlined criteria. One additional study was identified by manually searching the reference lists. In total, 23 articles were identified.

### Characteristics of the included studies

Table S3 in the supplementary material shows the key characteristics of the included studies. Most of the studies were conducted in America (*n* = 14), followed by Nigeria (*n* = 3), Pakistan (*n* = 2), Peru (*n* = 2), India (*n* = 1), and Kenya (*n* = 1). Only nine studies explicitly reported the type of community involved (9/23, 39.1%), with three studies including both rural and urban communities, three focusing exclusively on rural communities, and three concentrating solely on urban communities. These studies were performed in various designs, over half (*n* = 12) adopted uncontrolled pre-post design [19, 26–36], six used cluster-RCT (*n* = 6) [20, 37–41] and five employed non-RCTs (*n* = 5) [42–46]. These included studies focused on multiple vaccines, 10 targeted routine childhood vaccines [20, 28, 29, 33, 34, 36, 37, 39, 40, 46], 8 targeted HPV vaccines [19, 26, 30, 31, 41, 43–45], 4 targeted hepatitis B vaccines [27, 32, 38, 42] and 1 targeted a COVID-19 vaccine [35].

### CBPR component: establishment of CAPs

CAP establishment is the necessary and first step in CBPR projects, in which academic researchers and community stakeholders collaborate in an equal and cooperative partnership to share expertise [47]. Five studies used existing CAP networks [29, 31, 33, 38, 42], whereas 18 studies formulated new CAP networks [19, 20, 26–28, 30, 32, 34–37, 39–41, 43–46]. Only five studies detailed how to build CAP networks through meetings [19, 27], conversations [27], advocacy visits [19, 20, 27, 28], proactive invitations [19, 27, 28, 44], partnerships with institutes that have CAP networks [43] and support from the upper organisations of potential partners [27]. Advocacy visits and proactive invitations were the most common approaches. One study used five strategies to build a CAP network [27].

#### Types of community partners and their participation in different research phases

Table 1 displays the types of community partners. The included studies covered eight types of community partners, and health service institution-related non-government organisation (NGO) was the most common type (*n* = 21), followed by community service organisation-related NGO (*n* = 12), government agency (*n* = 9), and religious organisation (*n* = 5). Media agency was the least frequently encountered type of community partner (*n* = 1), followed by business agency (*n* = 2) and educational institution (*n* = 3). These studies involved different numbers of community partners, 69.6% of the studies (16/23) involved two types [19, 20, 29, 32, 36, 41, 44, 45] or three types of community partners [28, 30, 35, 38, 40, 42, 43, 46], and four studies involved four types of community partners [31, 33, 37, 39]. One study included six types of community partners [27], whereas another study included five types [34], and one study solely focused on one type of community partner [26].

**Table 1:** Types of community partners and their participation in different phases in each study

Study	The types of community partners								The research phases involving community partners							
	Community service organization–related NGO	Health service institution–related NGO	Religious organization	Government agency	Business agency	Education institution	Media agency	Community leader and representative	*N*	Subject recruitment	Intervention development	Intervention delivery	Data collection	Results interpretation	Findings dissemination	*N*
Bailey et al. [27]	✓	✓		✓	✓	✓	✓		6		✓	✓	✓			3
Ma et al. [42]	✓	✓	✓						3	✓	✓	✓	✓	✓	✓	6
Ma et al. [38]	✓	✓	✓						3	✓	✓	✓				3
Weir et al. [32]	✓	✓							2		✓	✓	✓			3
Levinson et al. [19]		✓						✓	2	✓		✓	✓			3
Abuelo et al. [26]		✓							1	✓		✓				2
Parra-Medina et al. [43]		✓				✓		✓	3	✓		✓	✓			3
Lee et al. [30]	✓	✓						✓	3	✓	✓					2
Paskett et al. [41]		✓						✓	2		✓	✓				2
Sanderson et al. [44]		✓						✓	2		✓	✓				2
Lennon et al. [31]	✓	✓		✓				✓	4	✓	✓	✓			✓	4
Ma et al. [45]		✓						✓	2	✓	✓	✓				3
Findley et al. [29]	✓	✓							2	✓	✓	✓	✓			4
Olayo et al. [46]		✓		✓				✓	3		✓	✓	✓	✓		4
Willis et al. [33]	✓	✓		✓				✓	4	✓	✓	✓	✓		✓	5
More et al. [39]	✓			✓	✓			✓	4			✓			✓	2
Habib et al. [37]		✓	✓			✓		✓	4			✓	✓			2
Bawa et al. [28]		✓		✓				✓	3		✓	✓	✓			3
Oyo-Ita et al. [40]		✓	✓					✓	3			✓	✓		✓	3
Akwataghibe et al. [34]	✓	✓	✓	✓				✓	5	✓	✓	✓				3
Khan et al. [20]		✓						✓	2	✓		✓				2
Suryadevara et al. [36]	✓			✓					2	✓		✓	✓			3
Marquez et al. [35]	✓	✓		✓					3	✓	✓	✓	✓			4
N	12	21	5	9	2	3	1	15		14	15	22	13	2	5	

Table 1 demonstrates the involvement of community partners in six distinct research phases, including subject recruitment, intervention development, intervention delivery, data collection, results interpretation, and findings dissemination. The phases of participation for community partners varied, and most community partners engaged in intervention delivery (*n* = 22), intervention development (*n* = 15), subject recruitment (*n* = 14), and data collection (*n* = 13). Few community partners engaged in finding dissemination (*n* = 5) and result interpretation (*n* = 2). The number of research phases involving community varied across the included studies, indicating different degrees of engagement among community partners. Only one study involved community partners in all six research phases [42], another engaged them in five phases [33], four studies covered four phases [29, 31, 35, 46], fewer than half (43.5%, *n* = 10) included community partners in three distinct phases [19, 27, 28, 32, 34, 36, 38, 40, 43, 45], and nearly one-third involved them in only two phases [20, 26, 30, 37, 39, 41, 44]. Details of the specific engagement phases of community in each study can be found in Table 1.

#### Maintenance of the CAP

**Fig. 2:**
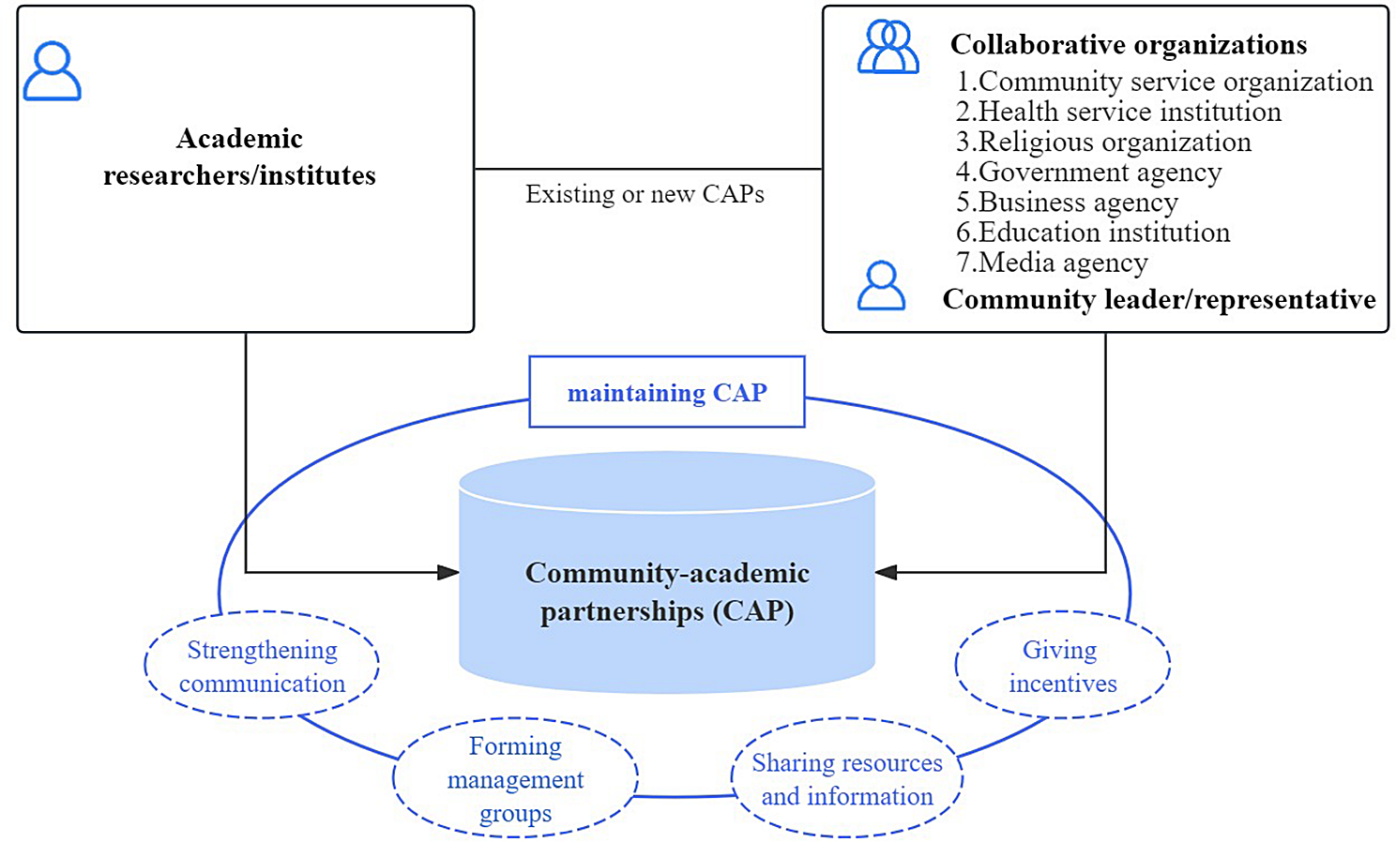
The CAP of CBPR approach for vaccination promotion. CAP was established between academic researchers/institutes and community partners. The eight types of community partners were community leaders/representatives and collaborative organizations, including community service organisation–related NGOs, health service institution–related NGOs, religious organisation, government agency, business agencies, education institutions, and media agencies. Four kinds of strategies could be used to maintain CAPs, which included strengthening communication, forming management groups, sharing resources or information, and providing incentives

Figure 2 presents the overall structure of CAPs and the strategies used for maintaining CAPs. These studies used four strategies to maintain CAPs by strengthening communication, forming management groups, sharing resources or information, and providing incentives. More than two-thirds of these studies (69.6%, 16/23) maintained the established CAPs by strengthening communication through meetings, dialogues, interviews, and community events between academic researchers and community partners during research processes [20, 27–29, 31, 33–35, 37–42, 44, 46]. More than a third of studies (39.1%, 9/23) maintained the established CAPs by forming management groups, such as steering committees, advisory boards, and subcommittees [27, 31, 33, 34, 38, 40, 42, 44, 46]. Nearly half of these studies (47.8%, 11/23) maintained the established CAPs by sharing resources or information between academic researchers and community partners [19, 27, 28, 31, 33, 34, 38–40, 42, 46]. Only one study maintained the established CAPs by providing incentives in the form of reimbursement of transportation costs to community partners [34].

### CBPR component: community capacity building by community partner training

Community capacity encompasses research literacy and vaccination service–related ability. Research literacy refers to community partners’ understanding of research stages and ability to collaborate with researchers effectively throughout the whole research process. Vaccination service–related ability includes knowledge of vaccines and immunisation, effective communication skills and self-efficacy in engaging with a target population. The community capacity building can be achieved by training community partners, and creating opportunities for academic researchers and community partners to express opinions, exchange ideas and share resources. The followings were the contents of training and delivery method, including the individuals responsible for training tasks, training sites, duration and frequency of the partner training sessions, and diverse training methods.

#### Contents of community partner training

Most of the included studies (82.6%, 19/23) conducted training for community partners [19, 20, 26–29, 32, 33, 35, 37–46], over half of the studies (56.5%, 13/23) provided detailed training contents [19, 20, 27–29, 32, 38–42, 44, 45], three studies demonstrated prepared training materials based on previous research experience or surveys [26, 35] and tools [40] and only one study used training materials verified by experienced community health officers [40].

Table 2 summarises the contents of the training materials for community partners. The contents contained four aspects: relevant knowledge of vaccination [19, 20, 26–29, 32, 35, 39–41, 44], and research project introduction [19, 20, 28, 29, 32, 38, 40, 42, 44] were the two most common aspects, followed by research literacy and skills [19, 20, 26, 29, 39, 40, 42] and service capacities and skills [29, 35, 39–41, 44].

**Table 2:** The contents of community partner training for community capacity building

Content domains	Number of studies	Specific description
Research project introduction	9	(1) research objectives, study protocol and project materials (2) community mobilization for the project (3) introduction of the project implementation (4) public health implications of the project
Vaccination relevant knowledge	12	(1) disease knowledge (2) vaccination knowledge or information (3) vaccination schedule
Research literacy and skills	7	(1) research plan (2) research methodology (3) follow-up strategies (4) data collection method (5) result presentation
Service capacities and skills	6	(1) communication skills (2) knowledge about leadership (3) knowledge and skills about good service

#### Delivery of community partner training

Nine studies specified individuals responsible for training tasks. Out of the studies, six relied solely on academic researchers to complete the training [19, 28, 40–42, 44], two relied exclusively on community partners [20, 39], and one study involved a collaborative effort between academic researchers and community partners (church leaders) to complete the training programmes [38]. These studies covered different stakeholders as trainees, and healthcare providers were the most commonly involved trainees [20, 26, 27, 32, 37, 40, 41, 44, 46], followed by community representatives [19, 20, 28, 33, 37, 39, 43, 46], religious staff [38, 40, 42], community-based organisation staff [29, 37], community traditional and religious leaders [40] and hired community coordinators [38].

These studies used different venues as training sites for partners, and local communities (such as health centre and town council hall) were the generally involved location [40, 44]. The duration of partner training varied from one hour to three days [19, 26, 40, 41, 44, 46], and the frequency of training sessions ranged from one to over 40 within a span of two years [19, 26, 35, 37, 40, 41, 44, 46]. One study performed partner training with various methods, including group discussions, brainstorming, role plays, case studies, and learning aids [40].

### CBPR component: development and implementation of community-based intervention

CBPR enabled collaborative and equitable partnerships between academic researchers and community partners throughout research phases [48]. Researchers recognized existing power differentials and addressed them by fostering trust and mutual respect, empowering the community, and tailoring their approaches to meet the community’s specific needs. Communities were engaged in decision-making processes ranging from identifying health issues to disseminating research findings. This efforts were aimed at optimally facilitating communication and decision-making, thereby promoting a more equitable distribution of power.

#### Development of community-based intervention

Twelve studies detailed strategies used to develop community-based interventions. Among these studies, six studies relied on baseline qualitative evaluation to inform the development of interventions [30, 34, 38, 42, 44, 46], four developed interventions based on community needs identified through baseline quantitative surveys [31–33, 35] and two utilised a combination of quantitative surveys and qualitative methods [38, 42]. The qualitative methods included interviews with religious or traditional leaders [38, 42, 46] and health professionals [44], focus groups with community representatives or leaders [30], and dialogues with community representatives or policymakers [34, 46]. The formulated interventions were updated and revised by community stakeholders [33, 34, 38, 42, 44] or validated for the refinement of the contents and structures of the interventions [30, 34].

#### Contents of community-based intervention

Table 3 shows the contents of the community-based interventions. The studies contained seven strategies, and health service support was the most frequently used strategy (*n* = 19), followed by follow-ups and home visits (*n* = 15), health education activities (*n* = 12), social marketing campaigns and community mobilisations (*n* = 9), interactive discussions (*n* = 9), vaccination reminders (*n* = 7), and financial or material incentives (*n* = 5).

**Table 3:** Contents of community-based intervention for vaccination promotion

Intervention strategies	Number of studies	Specific description
Health service supports	19	(1) clinical/service support (2) navigation assistance (3) referral service (4) making health service accessible by building community vaccination sites (5) updating health service facilities and increasing number of health workers
Follow-ups/home visits	15	(1) identifying unvaccinated participants, or arranging them to get vaccinated (2) management/follow ups after vaccination (3) feedback about results of medical examination and promotion of health-related behaviours (4) addressing knowledge and awareness about vaccination
Health education activities	12	*Educational contents* (1) introduction about the research project; (2) disease knowledge; (3) vaccination-related knowledge/information; (4) insurance and local health service–related information; (5) communication skills with different stakeholders; (6) testimonial of vaccinated/unvaccinated peers and physicians; (7) establishment or utilisation of social support system *Educational channels and ways* (1) websites; (2) telephone calls; (3) text messages; (4) mailing; (5) face-to-face (e.g., self-help learning, educational session); (6) interactive group discussion/activities *Type of educational materials* (1) printed materials; (2) videos
Social marketing campaigns and community mobilisations	9	(1) mobile billboards (2) public medias campaigns (3) automated text messages (4) door-to-door promotions (5) advertising at community activities or organisations
Interactive discussions	9	(1) addressing participants’ concerns about health/vaccination (2) increasing participants’ knowledge and information related to vaccination
Vaccination reminders	7	(1) reminder by mailings (2) reminder by automated devices (3) reminder by phone calls (4) reminder by emails (5) face-to-face reminder
Financial/material incentives	5	(1) free or low-cost vaccination (2) free or low-cost health service (3) gift (a book)

Table 4 illustrates the strategies utilised in each study for community-based interventions. Approximately half of the studies (47.8%, 11/23) implemented at least four types of strategies [20, 27–29, 31, 33, 35, 38, 42–44], six studies adopted three types [19, 34, 36, 39, 41, 46] and another six studies employed two types of strategies [26, 30, 32, 37, 40, 45].

**Table 4:** Contents of community-based interventions and the effects on vaccination rates

Study	The contents of interventions							Number of intervention contents	Increased vaccination rates
	Social marketing campaign/community mobilisation	Health education	Interactive discussion	Health service support	Financial /material incentive	Vaccination reminder	Follow-up		
Bailey et al. [27]	✓			✓	✓	✓	✓	5	49%
Ma et al. [42]		✓		✓	✓		✓	4	33%
Ma et al. [38]		✓		✓	✓		✓	4	66.4%
Weir et al. [32]				✓			✓	2	16.2%
Levinson et al. [19]			✓	✓			✓	3	92.9%
Abuelo et al. [26]				✓			✓	2	62.9%
Parra-Medina et al. [43]		✓		✓		✓	✓	4	29.7%
Lee et al. [30]		✓		✓				2	30%
Paskett et al. [41]	✓	✓				✓		3	4.5%
Sanderson et al. (2017)		✓	✓	✓		✓		4	-5.6%
Lennon et al. [31]	✓	✓				✓	✓	4	20.4%
Ma et al. [45]		✓	✓					2	65.5%
Findley et al. [29]		✓		✓	✓	✓	✓	5	34.5%
Olayo et al. [46]			✓	✓			✓	3	Measles OR: 1.144 Penta 3 OR: 1.073;
Willis et al. [33]	✓	✓	✓	✓				4	37%
More et al. [39]	✓		✓	✓				3	5.9%
Habib et al. [37]	✓			✓				2	7%; 9%
Bawa et al. [28]	✓		✓	✓			✓	4	OPV3: 38%; OPV: 30%; Penta3: 33%
Oyo-Ita et al. [40]		✓					✓	2	-2.9%
Akwataghibe et al. [34]	✓			✓			✓	3	27.8%
Khan et al. [20]	✓			✓	✓		✓	4	74%
Suryadevara et al. [36]			✓	✓		✓		3	17.3%
Marquez et al. [35]	✓	✓	✓	✓			✓	5	75.6%
N	10	12	9	19	5	7	15		

#### Implementation of community-based intervention

The included studies adopted various approaches to deliver interventions, and four studies integrated interventions into existing projects within community organisations [27, 29, 32, 36]. In most studies (73.9%, 17/23), interventions were delivered by researchers and partners [19, 27–29, 31, 33–38, 40–43, 45, 46], five studies solely relied on community partners [20, 26, 32, 39, 44], and one study conducted interventions by academic researchers alone [30]. Most interventions used a face-to-face delivery modality with various methods, such as group discussion and one-to-one interaction [19, 26–29, 31–33, 37–40, 42–46]. Some interventions were conducted through telephones [27, 36, 41, 43], text messages [30, 35, 45], and mail [27, 31, 41]. In some cases, interventions were tailored to participants’ preferences, allowing for the customisation of factors, such as the frequency and contents of text messages [30], individual or group settings, time, and location [33].

### CBPR component: outcome evaluation of community-based intervention

**Fig. 3:**
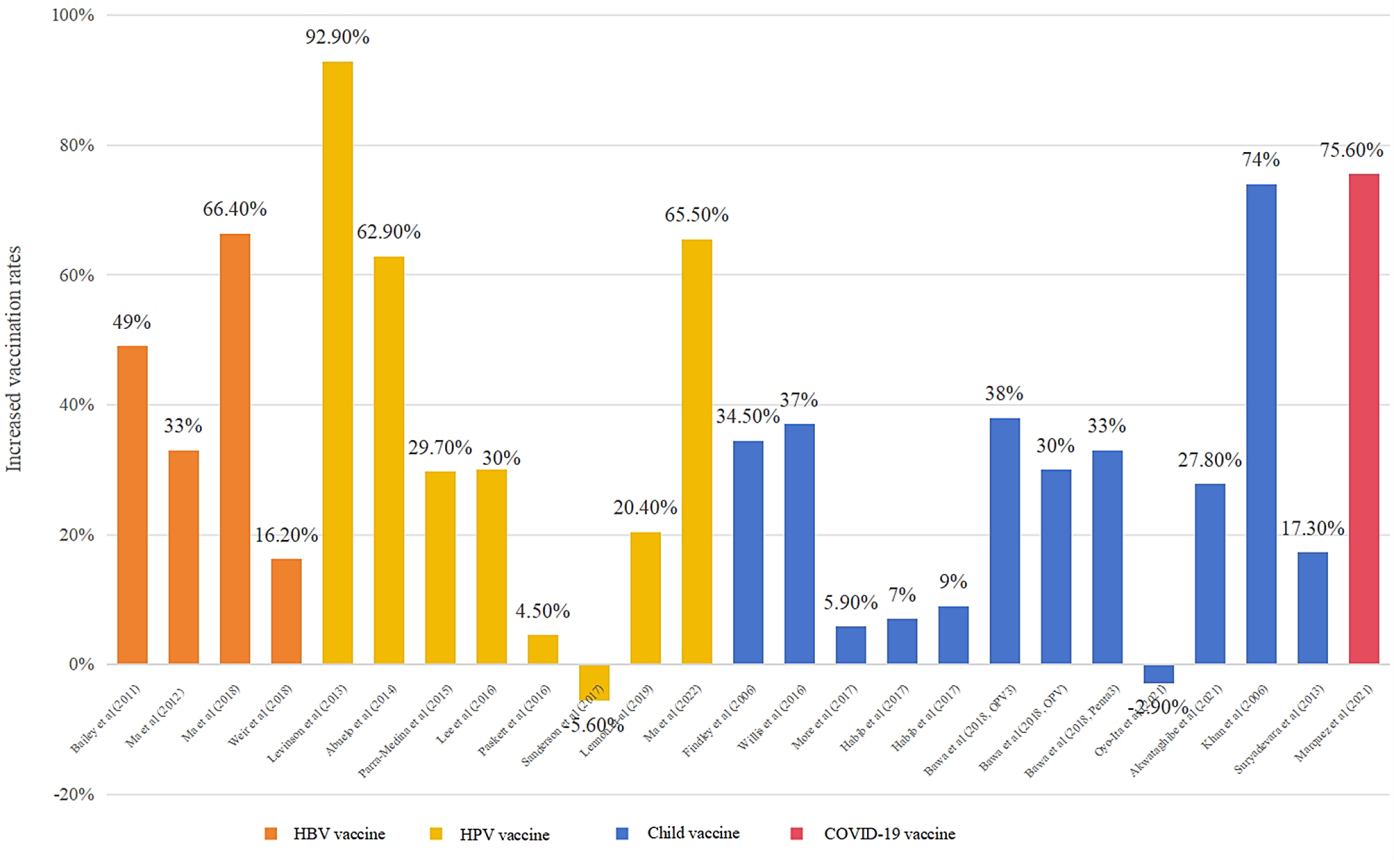
The increased vaccine rates after intervention in each study

Figure 3 visually demonstrates the increased vaccination rates observed in the intervention group in each study. The average increased vaccination rates for four types of vaccines were as follows: 75.6% for COVID-19 (*n* = 1), 41.2% for HBV (*n* = 4), 37.5% for HPV (*n* = 8), and 25.9% for childhood vaccines (*n* = 12). Most studies (*n* = 22) confirmed the positive effects of community-based interventions on increasing vaccination rates, and the highest observed vaccination rate for HPV reached 92.9% [19]. However, two studies did not find promising results. One study indicated that the community-based intervention did not lead to considerably different vaccination rates within the intervention group (51.8%) compared with the control group (54.7%) [40]. The other study showed similar results regarding improvements in the vaccine uptake of three-dose HPV (12.4% vs. 18.0%) [44]. Nearly half of these studies (*n* = 11) exhibited moderate increase in vaccine rate, which indicated at least 30% increase in vaccine rate after intervention [19, 20, 26–30, 33, 38, 42, 45]. A small number of studies (*n* = 3) exhibited small increase in vaccine rate, which exhibited less than 10% increase in vaccine rate after intervention [37, 39, 41].

Nine studies highlighted the potential of CBPR-based interventions in mitigating immunisation disparities through improving vaccination rates or expanding coverage within the vaccinated population [28–31, 33, 35, 38, 42, 46]. Three of them explicitly stated the objective of reducing health disparities associated with vaccination among high-risk underserved populations [29, 31, 42]. One study further pointed out the methodology for measuring the reduction of disparity by comparing immunization coverage rates with previous National Immunization Survey [29].

### Formulation of a CBPR-based implementation framework for vaccination promotion

**Fig. 4:**
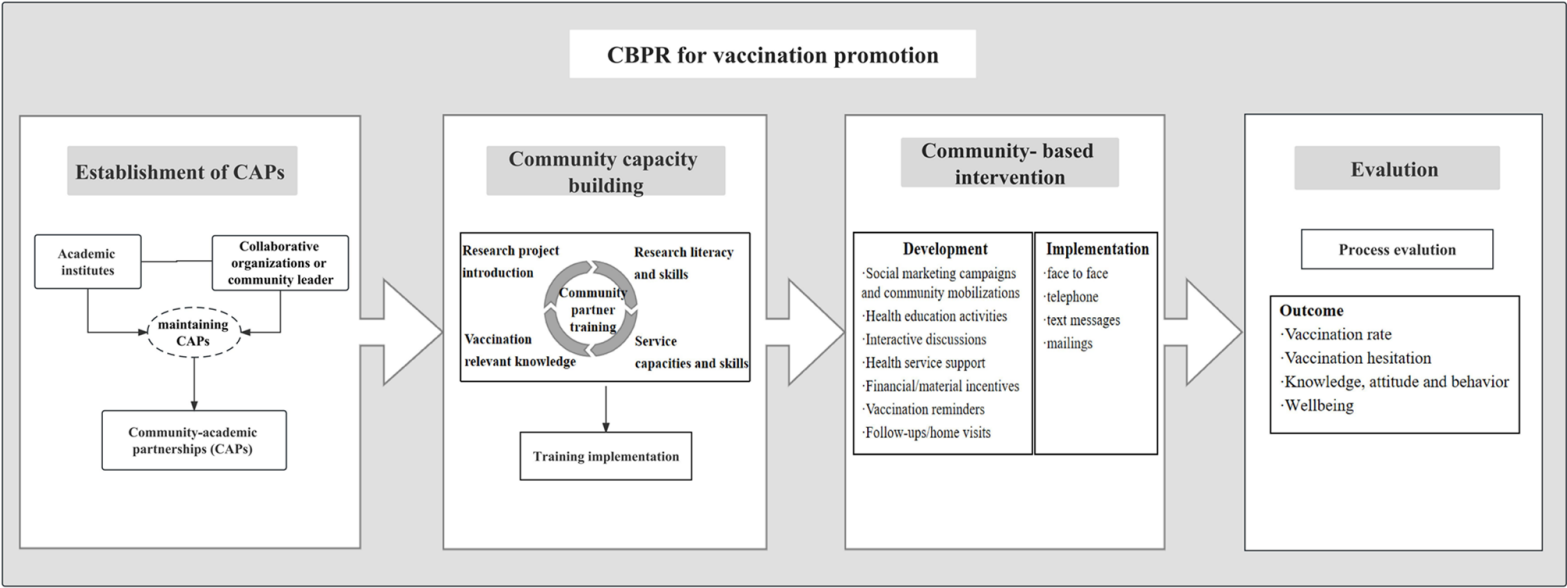
A CBPR-based implementation framework for vaccination promotion. A CBPR-based implementation framework for vaccination promotion was generated with four steps. (1) Establishment of CAP. CAP was established between academic researchers or institutes and community partners, and different kinds of strategies were used to maintain CAP. (2) Community capacity building by training community partners, including vaccination relevant knowledge, research project introduction, research literacy and skills as well as service capacities and skills. (3) Development and implementation of community-based intervention. Community-based intervention was developed with 7 intervention strategies, which involved health service supports, follow-ups/home visits, health education activities, social marketing campaigns, community mobilisation, interactive discussions, vaccination reminders and financial or material incentives. Community-based intervention was implemented through 4 delivery strategies, which involved face-to-face, telephone, text message, and mailing. (4) Outcome evaluation mainly focused on vaccine rate in these studies. Future studies could incorporate broader vaccination outcomes, such as vaccine hesitancy, long-term outcomes, and well-being

Based on the three domains of the CBPR conceptual model, four components of CBPR approach and evidence summarised above, an implementation framework was developed (Fig. 4), which refines the elements and steps of a CBPR approach for vaccination promotion. The details of the partnership dynamics of CAP is displayed in Fig. 2. Four strategies were implemented to reinforce the established CAP, which involved strengthening communication, forming management groups, sharing resources or information, and providing incentives.

## Discussion

CBPR has emerged in the last century as an action-oriented research paradigm to develop and implement community-based interventions, which can redress power imbalance, facilitate mutual benefits, and promote knowledge translations for all stakeholders [47, 49, 50]. Community participation ensures the credibility of a research project and enhances its usefulness by aligning it with what the community perceives as important social and health goals. This approach is characterised by equitable collaboration between academic researchers and community stakeholders in the whole research process and focuses on translating research findings into practical application within the community and is widely used for community health equity and improvement [51, 52]. Despite the potential of CBPR-based efforts to address immunisation gaps, their consistent replication and widespread success have yet to be demonstrated, as prevailed by the persistent disparities in immunisation uptake. This may be related to the discrepant and inconclusive core elements and practical steps of CBPR in the field of vaccination promotion [50]. Therefore, this scoping review was performed to summarise the components of CBPR approaches from the aspects of CAP establishment, community capacity building, intervention development and implementation, and outcome evaluation and to generate a framework to guide the utilisation of CBPR approaches in the process of vaccination promotion.

### Establishment of CAPs

As the critical component of CBPR, a CAP is characterised by equitable control, relevance to the community of interest, specific aims, and the active involvement of community members and academic researchers [53]. The establishment of CAP converged to deliver interventions and facilitate the translation of information into practice [54], varied in organisational structures and power allocation [55]. Except the five studies with pre-existing CAP networks, the studies prioritised the establishment of CAPs as the initial step in their CBPR programmes. The findings indicated that academic researchers were found to be the most common initiators in the establishment of CAPs, consistent with the findings of previous studies [53, 56]. The role of academic researchers as primary initiators can be attributed to the community partners’ limited awareness of health-related questions and their mistrust of academic research [57]. The establishment of a CAP is time-consuming and resource intensive, whereas trust is foundational to the sustainability of a CAP [58]. Further efforts are warranted to build trust in the establishment of a partnership. The included studies utilised the same methods for establishing CAPs as those used in previous studies. These methods involved meetings, conversations, advocacy visits and active invitations [59, 60]. The meetings, conversations and advocacy visits could help identify key issues and community needs to ensure buy-in from essential stakeholders and constituencies [19, 27]. Moreover, inviting active support from higher-level organisations greatly facilitated the establishment of the CAPs [61]. Thus, identifying influential groups or prominent individuals who can increase the likelihood of collaboration with targeted partners is promising method for future research on CAPs.

CAP enabled a collaborative partnership among diverse stakeholders, which followed the principles of equal participation in CBPR approach and recognised the strengths of stakeholders in community [62]. However, previous studies rarely reported characteristics, such as membership numbers or duration of CAP [53]. By contrast, the findings of this scoping review have provided additional insights into these aspects. The collaborative partnership consisted of a diverse group of members, which included community service organisation–related NGOs, health service institution–related NGO, religious organisation, government agency, business agency, education institution, and media agency. The types of partners and the research phases of participation varied with the unique context of each community and fluctuated across the phases of the research project. This variation in the composition of the CAP networks highlights the importance of tailoring an approach for network construction based on the specific needs and context of a community being served [63].

The types of community partners and their participation in various research phases were similar to those of previous literature [56, 64]. Community partners were more frequently involved in intervention development and delivery, participant recruitment, and data collection, while researchers were involved in traditional research activities. This division of tasks likely reflected a logical distribution of roles based on respective areas of expertise [55]. In other words, the background features of community partners influenced their involvement throughout the research process. Community partners with community knowledge and expertise actively contributed to the intervention development and implementation. However, their limited academic literacy and research leadership skills restricted their participation in data collection and results interpretation. Future studies can undertake multiple initiatives to train community partners with regard to research literacy. These initiatives may facilitate community partners to be engaged equitably throughout the research process.

Four strategies can be implemented to reinforce the partnership dynamics in the established CAP, which involved strengthening communication, forming management groups, sharing resources or information, and providing incentives. A successful CBPR partnership could be strengthened by the effective communication through regular group meetings [42]. Previous study also demonstrated that meetings, dialogues, interviews, and community events were common strategies and can effectively facilitate the maintenance of CAPs [56]. Other studies inversely validated the importance of communication in CAP, which demonstrated that inadequate communication was a common impediment to CAP establishment [65, 66]. Forming management groups, such as steering committee that included both community and research representatives, could enhance coordination, clarify roles and responsibilities, and facilitate consensus building in the planning stage or the intervention process [33, 46]. Financial incentives were the least frequently utilised strategy (*n* = 5) among the included studies. The lack of financial incentives was a common hindering factor to the construction of CAP, as verified in a systematic review [53]. Therefore, future studies need to clarify communication channels and ensure financial incentives to mobilise stakeholders joining CAPs.

### Community capacity building by community partner training

CBPR relies on iterative processes to generate and build knowledge and is an ongoing co-learning effort, in which researchers and community partners work collaboratively to build capacity to address health issues [67]. Community partner training is a major avenue of community capacity building, providing necessary knowledge and skills for effectively implementing and sustaining CBPR projects. Community partners equipped with community expertise and academic skills are equitably and deeply engaged in the research process, which promotes collaboration between researchers and community partners and bridges the connection between research and community practice [68]. Among the included studies, only three studies developed the training materials based on previous investigations. For instance, one study developed training contents based on a prior survey of community members’ vaccine attitudes and preferences [35]. One study designed the training by the research team, validated the contents with experienced community health officers, and conducted a pilot testing to ensure the effectiveness of the training [40]. The limited number of studies developing training based on evidence deviated from the iterative development or the theoretical model–based design approach employed in previous studies for creating the contents of training programmes [69, 70]. The integration of validated scientific evidence into training contents likely enhanced their acceptability and applicability, thereby resulting in effective capacity building efforts. The present review examined the value of community participation in refining training materials and designing training courses, which facilitated the development of training programmes to meet the aspirations of trainees and the needs of local communities [71, 72]. Health knowledge and research literacy were identified as the two most prevalent topics in community partner training. This finding was consistent with a study showing that relevant health and research knowledge were the two main topics in community health worker training [73].

The included studies exhibited wide variations in training delivery in terms of trainers, duration, frequencies, and methods. Among nine studies that specified individuals responsible for training tasks, academic researchers were the most common type (66.7%, 6/9). The exclusion of community partners in training challenged the acceptability and effectiveness of training by ignoring stakeholders’ preferences and community needs. A qualitative study among community stakeholders revealed the importance of adopting a collaborative approach to incorporate community stakeholders and researchers to align training competencies [74]. The included studies conducted training with varied durations and frequencies. Only one study conducted refresher training four months since the start of the intervention [44]. The importance and impact of training diminished over time [75, 76]. All these studies underscored the importance of conducting multiple training sessions at an appropriate frequency. Regarding the training method, limited information was disclosed in the included studies (*n* = 1). Previous studies adopted diverse methods in the training of community partners through didactic presentation, group discussion, and role plays, which may help effectively disseminate and implement curricula to health care workers [77].

### Development and implementation of community-based intervention

Many included studies utilised specific methods to develop community-based interventions based on community needs and previous evidence. Quantitative surveys and qualitative methods were employed to evaluate stakeholder claims and community needs and clarify partners’ views and preferences on community health questions. Previous studies highlighted the importance of community needs and practice evidence in intervention development [78, 79], which could ensure the cultural adaptability and community relevance of interventions and increase the acceptability and feasibility of interventions. This review suggested that most studies engaged community partners in intervention implementation, and some interventions were tailored to the preferences of participants. Such efforts can enhance the receptivity of interventions because community partners were familiar with the communities. In addition, this allowed community partners to deliver interventions to participants in the most suitable and acceptable manner and increase their effectiveness.

### Outcome evaluation of community-based intervention

The findings of this scoping review indicated the success of CBPR in vaccination promotion, whereby most included studies supported the effectiveness of community-based intervention on vaccine rate. The outcome evaluation of CBPR programmes in the included studies primarily focused on vaccination rates. Future studies could explore broader vaccination outcomes, including vaccine hesitancy, vaccine confidence, and long-term outcomes, such as well-being and disease incidence. By examining these broad outcomes, a more comprehensive understanding of the impacts of CBPR programmes on vaccination and public health can be gained.

Improving the vaccination rate among vulnerable groups could contribute to addressing disparities in vaccine-associated diseases. The involvement of the community in the planning and implementation processes, fully integrated within routine organisational programs, facilitates the equitable distribution of resources and promotes the sustainability of disparities elimination efforts. Therefore, it is advisable for public health officials to undertake and implement CBPR-based interventions to enhance health equity among populations affected by disparities.

### Limitations

The review had limitations. Despite the inclusion of studies conducted in diverse countries for a long period, evidence yielded in this review may not apply to diverse demographic groups in various communities. Comparing the effects of vaccination promotion across different types of vaccinations and various research phases involving communities is challenging due to limitations like different study designs, data heterogeneity, significant variability in vaccine types, and diverse targeted populations, all of which further complicate the interpretation of the results. In addition, given that the focus was directed to the elements and steps of CBPR approaches to vaccination promotion, other problems, such as promoters and barriers in the development and implementation of CBPR programmes were not extensively emphasised.

## Conclusion

This scoping review firstly summarised the core elements and practical steps of CBPR and generated an implementation framework, specifically in the context of vaccination promotion. The results highlighted the success and potential of utilising CBPR for improving vaccination rates and indicated that researchers and community practitioners to further expand theoretical orientations and methodological toolkit associated with CBPR. The CBPR-based implementation framework, including CAP establishment, community capacity building, intervention development and implementation, and outcome evaluation, can serve as a framework for future vaccination promotion programmes involving community stakeholders and research professionals to facilitate equitable vaccination access for diverse populations.

## Electronic supplementary material

Below is the link to the electronic supplementary material.

Supplementary Material 1

## Data Availability

All data generated or analysed during this study are included in the supplementary information files of this article.

## Abbreviations

CAP: Community Academic Partnership
CBPR: Community-based Participatory Research
NGOs: Non Government Organisations
PICOS: Population, Intervention, Comparison, Outcome, Study design
RCT: Randomised Controlled Trial
