# Supplementary material for: Community-based participatory research (CBPR) approaches in vaccination promotion: a scoping review
Source: Int J Equity Health. 2024 Nov 5;23:227. doi: 10.1186/s12939-024-02278-1 (PMC11539765; doi:10.1186/s12939-024-02278-1)
Supplement: Supplementary file 1 — Supplementary Material 1 [file 12939_2024_2278_MOESM1_ESM.docx]

***Supplementary material Table List***

*Table S1 Preferred Reporting Items for Systematic reviews and Meta-Analyses extension for Scoping Reviews (PRISMA-ScR) Checklist*

*Table S2 Search strategies and search results of electronic databases*

*Table S3 Characteristics of included studies*

**Table S1** Preferred Reporting Items for Systematic reviews and Meta-Analyses extension for Scoping Reviews (PRISMA-ScR) Checklist

| **SECTION** | **ITEM** | **PRISMA-ScR CHECKLIST ITEM** | **REPORTED ON PAGE #** |
| --- | --- | --- | --- |
| **TITLE** | | | |
| Title | 1 | Identify the report as a scoping review. | 1 |
| **ABSTRACT** | | | |
| Structured summary | 2 | Provide a structured summary that includes (as applicable): background, objectives, eligibility criteria, sources of evidence, charting methods, results, and conclusions that relate to the review questions and objectives. | 2-3 |
| **INTRODUCTION** | | | |
| Rationale | 3 | Describe the rationale for the review in the context of what is already known. Explain why the review questions/objectives lend themselves to a scoping review approach. | 4-5 |
| Objectives | 4 | Provide an explicit statement of the questions and objectives being addressed with reference to their key elements (e.g., population or participants, concepts, and context) or other relevant key elements used to conceptualize the review questions and/or objectives. | 5 |
| **METHODS** | | | |
| Protocol and registration | 5 | Indicate whether a review protocol exists; state if and where it can be accessed (e.g., a Web address); and if available, provide registration information, including the registration number. | Not applicable |
| Eligibility criteria | 6 | Specify characteristics of the sources of evidence used as eligibility criteria (e.g., years considered, language, and publication status), and provide a rationale. | 6-8 |
| Information sources* | 7 | Describe all information sources in the search (e.g., databases with dates of coverage and contact with authors to identify additional sources), as well as the date the most recent search was executed. | 6-7 |
| Search | 8 | Present the full electronic search strategy for at least 1 database, including any limits used, such that it could be repeated. | 6-7 |
| Selection of sources of evidence† | 9 | State the process for selecting sources of evidence (i.e., screening and eligibility) included in the scoping review. | 6-7 |
| Data charting process‡ | 10 | Describe the methods of charting data from the included sources of evidence (e.g., calibrated forms or forms that have been tested by the team before their use, and whether data charting was done independently or in duplicate) and any processes for obtaining and confirming data from investigators. | 8 |
| Data items | 11 | List and define all variables for which data were sought and any assumptions and simplifications made. | 8 |
| Critical appraisal of individual sources of evidence§ | 12 | If done, provide a rationale for conducting a critical appraisal of included sources of evidence; describe the methods used and how this information was used in any data synthesis (if appropriate). | Not applicable |
| Synthesis of results | 13 | Describe the methods of handling and summarizing the data that were charted. | 8 |
| **RESULTS** | | | |
| Selection of sources of evidence | 14 | Give numbers of sources of evidence screened, assessed for eligibility, and included in the review, with reasons for exclusions at each stage, ideally using a flow diagram. | 9 |
| Characteristics of sources of evidence | 15 | For each source of evidence, present characteristics for which data were charted and provide the citations. | 9 |
| Critical appraisal within sources of evidence | 16 | If done, present data on critical appraisal of included sources of evidence (see item 12). | Not applicable |
| Results of individual sources of evidence | 17 | For each included source of evidence, present the relevant data that were charted that relate to the review questions and objectives. | 10-15 |
| Synthesis of results | 18 | Summarize and/or present the charting results as they relate to the review questions and objectives. | 10-15 |
| **DISCUSSION** | | | |
| Summary of evidence | 19 | Summarize the main results (including an overview of concepts, themes, and types of evidence available), link to the review questions and objectives, and consider the relevance to key groups. | 15-20 |
| Limitations | 20 | Discuss the limitations of the scoping review process. | 20 |
| Conclusions | 21 | Provide a general interpretation of the results with respect to the review questions and objectives, as well as potential implications and/or next steps. | 21 |
| **FUNDING** | | | |
| Funding | 22 | Describe sources of funding for the included sources of evidence, as well as sources of funding for the scoping review. Describe the role of the funders of the scoping review. | 23 |

| **Table S2** Search strategies and search results of electronic databases | |
| --- | --- |
| **DATABASES** | **Results** |
| **PubMed** | 2,022 |
| ((((Vaccines [Mesh]) OR Vaccination [Mesh]) OR Vaccination Coverage [Mesh]) OR (((vaccin*[Title/Abstract]) OR (immuniz*[Title/Abstract]))  OR (immunis*[Title/Abstract]))) AND ((("Community-Based Participatory Research"[Mesh]) OR "Community Participation"[Mesh]) OR  (((((((((((((((((((((((((((((((((((((Community Based Participatory Research [Title/Abstract]) OR (Participatory Research, Community-Based [Title/Abstract])) OR (Consumer-Driven Community-Based Research [Title/Abstract])) OR (Community-Based Research, Consumer-Driven [Title/Abstract])) OR (Community-Based Research, Consumer-Driven [Title/Abstract])) OR (Consumer Driven Community Based Research [Title/Abstract])) OR (Consumer-Driven Community-Based Research [Title/Abstract])) OR (Research, Consumer-Driven Community-Based [Title/Abstract])) OR (Research, Consumer-Driven Community-Based [Title/Abstract])) OR (Participation, Community [Title/Abstract])) OR (Community Involvement [Title/Abstract])) OR (Community Involvements [Title/Abstract])) OR (Involvement, Community [Title/Abstract])) OR  (Involvements, Community [Title/Abstract])) OR (Consumer Participation [Title/Abstract])) OR (Participation, Consumer [Title/Abstract])) OR (Consumer Involvement [Title/Abstract])) OR (Consumer Involvements [Title/Abstract])) OR (Involvement, Consumer [Title/Abstract])) OR  (Public Participation [Title/Abstract])) OR (Participation, Public [Title/Abstract])) OR (Community Action [Title/Abstract])) OR (Action, Community [Title/Abstract])) OR (Actions, Community [Title/Abstract])) OR (Community Actions [Title/Abstract])) OR (participatory research [Title/Abstract])) OR (participatory action research [Title/Abstract])) OR (participatory approach [Title/Abstract])) OR (community engag*[Title/Abstract])) OR (community invovl*[Title/Abstract])) OR (community participat*[Title/Abstract])) OR (community stakeholder*[Title/Abstract])) OR (community partner*[Title/Abstract]))) OR (community-centered activit*[Title/Abstract])) OR (community-based collaborat*[Title/Abstract])) OR (academic community [Title/Abstract]))) |  |
| Embase | 784 |
| Web of Science | 4,479 |
| Cochrane Library | 1,272 |

**Table S3** Characteristics of included studies

| **Author (year)** | **Country** | **Study design** | **Participants** | **Intervention** | **Comparation** | **Vaccine types** | **Vaccination outcomes** |
| --- | --- | --- | --- | --- | --- | --- | --- |
| Ma (2018) | USA | Cluster RCT | Korean American aged 18 years or above (N=1834) | Health education and discussion  Health service support | Health education about general cancer and health issues | HBV | Full immunization (three doses)  (1) Control group: 17.6% (3/17)  (2) Intervention group: 84% (279/332) |
| Ma (2012) | USA | Non-RCT | Korean American aged 18 years or over (N=330) | Health education and discussion  Health service support | Delayed intervention | HBV | HBV screening and vaccination rate:   1. Control group: 39.8%; 0%   (2) Intervention group: 95.8%; 33%(109/330) |
| Bailey (2011) | USA | Uncontrolled pre-post study | Asian/Pacific islanders (N=1962) | SMCs/CMs  Follow ups and reminders  Health service support | NA | HBV | Full immunization (three doses)  (1) Pre-intervention: 0%  (2) Post-intervention: 49% (981/1962) |
| Weir (2018) | USA | Uncontrolled pre-post study | People aged 18-70 years (N=1914) | Follow ups and reminders | NA | HBV | Partial immunization (at least 1 dose)  (1) Pre-intervention: 11.7% (111/929)  (2) Post-intervention: 27.9% (275/ 985) |
| Paskett (2016) | USA | Cluster RCT | Caregivers (N= 337)  Adolescents aged 9-17 years  (N= 337) | SMCs/CMs  Health education and discussion  Follow ups and reminders | Health education about influenza and vaccine | HPV | First shot immunization  (1) Control group: 3.2% (4/125)  (2) Intervention group: 7.7% (10/130) |
| Levinson (2013) | Peru | Uncontrolled pre-post study | Caregivers (N= 323)  Girls aged 10-13 years (N=352) | Health education and discussion Follow ups and reminders  Health service support | NA | HPV | Full immunization (three doses)  (1) Pre-intervention: 0%  (2) Post-intervention: 92.9% (300/352) |
| Abuelo (2014) | Peru | Uncontrolled pre-post study | Caregivers (N= 320)  Girls aged 10-13 years (N=318) | Follow ups and reminders  Health service support | NA | HPV | Full immunization (three doses)  (1) Pre-intervention: 0%  (2) Post-intervention: 62.9% (200/318) |
| Lee (2016) | USA | Uncontrolled pre-post study | Women aged 21-29 years（N=30） | Health education and discussion  Health service support | NA | HPV | Partial immunization (1 dose)  (1) Pre-intervention: 0% (0/30)  (2) Post-intervention: 30% (9/30) |
| Lennon (2019) | USA | Uncontrolled pre-post study | Parents/ caregivers (N=118)  Adolescents aged 13-17 years (N=118) | SMCs/CMs  Health education and discussion  Follow ups and reminders | NA | HPV | UTD immunization  (1) Pre-intervention: 25.4% (30/118)  (2) Post-intervention: 45.8% (54/ 118) |
| Parra-Medina (2015) | USA | Non-RCT | Hispanic women (N=372)  Daughter aged 11-17 years (N=372) | Health education and discussion Follow ups and reminders  Health service support | HPV vaccine educational brochures | HPV | Full immunization (three doses)  (1) Control group: 42.5%  (2) Intervention group: 72.2% |
| Sanderson (2017) | USA | Non-RCT | Mothers (N=305)  Adolescent aged 9-18 years  (N= 408) | Health education and discussion Follow ups and reminders | Usual community care | HPV | Full immunization (three doses or more)  (1) Control group: 18.0 % (30/167)  (2) Intervention group: 12.4 % (24/194) |
| ﻿Ma (2022) | USA | Non-RCT | Chinese American caregivers (N=180)  Adolescent aged 11-18 years (N=180) | Health education and discussion Follow ups and reminders | General health education | HPV | Full immunization (three doses)  (1) Control group: 0% (0/70)  (2) Intervention group: 65.5% (72/110) |
| Findley (2006) | USA | Uncontrolled pre-post study | Caregivers (N= no information)  Children aged 19-35 months (N=1502) | Health education and discussion Follow ups and reminders  Health service support | NA | Immunization antigen series 4:3:1:3:3 | UTD immunization  (1) Pre-intervention: 46%  (2) Post-intervention: 80.5% |
| Khan (2006) | Pakistan | Cluster RCT | Children aged 2 to 16 years (N=21059) | Information dissemination, training and immunization campaign | NA | ViPS  HAV | Final vaccine coverage: 74% |
| More (2017) | India | Cluster RCT | Caregivers (N= no information)  Children aged 12-23 months (N=2251) | SMCs/CMs  Health education and discussion  Follow ups and reminders | Routine immunization | BCG  DPT3  Polio  HBV3  Measles | Full immunization  (1) Control group: 61.9% (708/1143)  (2) Intervention group: 67.8% (751/1108) |
| Habib (2017) | Pakistan | Cluster RCT | Caregivers (N= no information)  Children aged 1 month to 5 years (N= 87984) | SMCs/CMs  Health service support | Routine immunization | OPV  EPI | (1) OPV  Control group (arm A): 75%  Intervention group (arm B): 82%  Intervention group (arm C): 84%  (2) EPI  Control group (arm A): 25%  Intervention group (arm B): 32%  Intervention group (arm C): 34% |
| Oyo-Ita (2021) | Nigeria | Cluster RCT | Caregivers (N= 2598)  Children aged 0-23 months (N=2598) | Health education and discussion Follow ups and reminders | Routine immunization | BCG  OPV  Penta 3  PCV  Measles  Yellow fever | UTD immunization  (1) Control group: 54.7% (697/1274)  (2) Intervention group: 51.8% (661/1276) |
| Willis (2016) | USA | Uncontrolled pre-post study | Caregivers (N=no information)  Children aged 9-35 months (N=189) | SMCs/CMs  Health education and discussion | NA | ﻿Immunization antigen series 4:3:1:3:3:1 | UTD immunization  (1) Pre-intervention: 45%  (2) Post-intervention: 82% |
| Bawa (2018) | Nigeria | ﻿ Uncontrolled pre-post study | Mothers (N= no information)  Children aged under 1 year (N=222879) | SMCs/CMs  Follow ups and reminders  Health service support | NA | OPV3  OPV  Penta 3 | (1) OPV3 (children under 1year)  Pre-intervention: 23%  Post-intervention: 61%  (2) OPV (children aged 1-5 years)  Pre-intervention: 60%  Post-intervention: 90%  (3) Penta 3 (children under 1year)  Preintervention: 22%  Postintervention: 55% |
| Akwataghibe (2021) | Nigeria | Uncontrolled pre-post study | Caregivers (N=282)  Children aged 9 - 59 months  (N= 340) | SMCs/CMs  Follow ups and reminders  Health service support | NA | DPT3  Penta3  Measles  Yellow fever | Full immunization  (1) Pre-intervention: 19.8% (34/172)  (2) Post-intervention: 47.6% (80/168) |
| Suryadevara (2013) | USA | Uncontrolled pre-post study | Caregivers (N= 630)  Children aged under 17 years (N=1531) | Health education and discussion, follow ups and vaccination reminders | NA | PCV-13  TIV  HPV Meningococcal | Full immunization  (1) Pre-intervention: 28.2% (416/477)  (2) Post-intervention: 45.5 % (672/1477) |
| Olayo (2014) | Kenya | Non-RCT | Eight community health units (immunization trageting children) | Community Health Strategy based on the policy implementation guidelines. | NA | Measles  Penta 1  Penta 3 | Vaccination rates:   1. Measles: OR=1.144 (*P*=0.0335) 2. Penta 1: OR=0.662 (*P*=0.25) 3. Penta 3: OR=1.073 (*P*=0.8062） |
| Marquez (2021) | USA | Uncontrolled pre-post study | People aged over 16 years (N=12103) | SMCs/CMs  Health education and discussion Health service support | NA | COVID-19 | UTD immunization (2 doses)  (1) Pre-intervention: 0% (0/12103)  (2) Post-intervention: 75.6% (9152/12103) |

**Abbreviation**:

**HAV**: hepatitis A virus **HBV**: hepatitis B virus **HPV**: human papillomavirus **OPV3**: oral polio vaccine (three dose) **IPV**: inactivated polio vaccine **PCV13**: pneumococcal conjugate vaccine-13 vaccine **TIV**: trivalent influenza vaccine **MMR vaccine**: measles-mumps-rubella vaccine **Penta 3**: Diphtheria, Tetanus, Pertussis, hepatitis b; Haemophilus influenzae b (three doses) **DPT3**: Diphtheria, Tetanus, Pertussis (three doses) **BCG**: Bacillus Calmette-Guerin vaccine **Routine EPI vaccines**: BCG, Penta3, measles vaccine **Immunization antigen series 4:3:1:3:3**: 4 diphtheria-tetanus-pertussis, 3 polio, 1 measles-mumps-rubella, 3 Haemophilus influenza b, 3 Hepatitis B **Immunization antigen series 4:3:1:3:3:1**: 4 diphtheria-tetanus-acellular pertussis; 3 polio; 1 measles-mumps-rubella; 3 hepatitis b; 3 Haemophilus influenzae b; 1 varicella **NA**: not applicable **SMCs/CMs**: social marketing campaigns or community mobilizations **ViPS:** Vi polysaccharide
